# The Retentive Strength of Zirconium Oxide Crowns Cemented by Self-Adhesive Resin Cements before and after 6 Months of Aging

**DOI:** 10.3390/ma13183998

**Published:** 2020-09-09

**Authors:** Shifra Levartovsky, Lilac Cartier, Maya Brand, Jaron John Blasbalg, Raphael Pilo

**Affiliations:** Department of Oral Rehabilitation, The Maurice and Gabriela Goldschleger School of Dental Medicine, Sackler Faculty of Medicine, Tel Aviv University, Tel Aviv 6997801, Israel; lilac.cartier@gmail.com (L.C.); maya.brand@mail.huji.ac.il (M.B.); blasbalg@gmail.com (J.J.B.); rafipilo@tauex.tau.ac.il (R.P.)

**Keywords:** retentive strength, zirconium oxide, self-adhesive resin cements, aging

## Abstract

The aim of this study was to evaluate the retentive strength of zirconium oxide (yttria-stabilized tetragonal zirconia polycrystals (Y-TZP)) crown-copings treated by combined mechanical and chemical treatments and cemented by four types of self-adhesive resin cements (SARCs) to human prepared teeth, before and after six months of aging in water and thermocycling. A total of 120 molar teeth were mounted, prepared using a standardized protocol and digitally scanned, and Y-TZP copings were produced. Teeth were randomly assigned to four SARC groups. Prior to cementation, the intaglio surfaces of all crowns were sandblasted and then coated with Z-Prime™ Plus (Bisco Dental, Schaumburg, IL, USA). Post cementation, each cement group was subdivided into aged and non-aged groups. After aging, the cemented assemblies were tested for retentive strength using a universal testing machine. Failure analysis was conducted by inspecting all matched debonded surfaces of the teeth and crowns at 3× magnification. Aging treatment did not affect the retentive strength of the Y-TZP crown-copings (*p* = 0.918). The interaction between cement and aging was statistically significant (*p* = 0.024). No significant differences in the retentive strengths between the different SARCs were observed pre-aging (*p* = 0.776), whereas post-aging, Panavia SA (PAN; Kuraray Dental Co Ltd., Osaka, Japan) showed significantly higher strength than RelyX U-200 (RU200; 3M ESPE, Seefeld, Germany). The predominant failure mode was adhesive between the cement and dentin, followed by mixed mode failure.

## 1. Introduction

The increased demand for esthetic restorations has led to a tremendous shift toward all-ceramic materials as an alternative to metal-ceramic restorations. With the development of computer-assisted design/computer-aided manufacturing (CAD/CAM) systems, zirconium oxide (Y-TZP) ceramics have gained popularity due to their superiority to metal-ceramic restorations esthetically, mechanical stability and biocompatibility [1,2]. High flexural strength (900–1200 MPa) and high fracture resistance (more than 2000 N) make zirconium oxide ceramics suitable for fixed partial dentures (FPDs) supported by teeth and implants [3].

The long-term clinical success of Y-TZP FDPs is determined not only by the mechanical strength of the material but also by the cementation process, specifically by the adhesion of the cements to both the tooth structure and the restoration material [4]. Y-TZP crowns can be cemented to human dentin by conventional cements (zinc phosphate cement and glass ionomer), adhesive resin cements in total-etch (TE) or self-etch (SE) modes and self-adhesive resin cements (SARCs). As a result of adhesive bonding systems and resin cements, Y-TZP crowns have demonstrated increased retentive capabilities and fracture resistance of the restorative materials [5]. TE and SE systems require one or more steps before application, with increased chair time and high technique sensitivity [6], whereas SARCs, with their acidic methacrylate monomers, do not require preliminary steps and provide a bond strength similar to that of other adhesive systems [7,8,9].

Palacios et al. [10] demonstrated that there were no statistically significant differences in the retentive strength of luting agents for zirconium oxide-based crowns with the following cements: composite resin cement with adhesive agent (Panavia F 2.0 and ED Primer A & B), a resin-modified glass-ionomer cement (Rely X Luting) and a SARC (RelyX Unicem). Ernst et al. [11] studied the retentive strength of four resin–cement systems—a compomer, a glass-ionomer cement, a resin-modified glass-ionomer cement and a SARC—for luting zirconium oxide ceramic crowns and found no significant difference in their retentive quality. Moreover, the retentive force between Y-TZP crowns and tooth dentin was found to be affected by the surface conditioning of the Y-TZP ceramics more than by the cement type [12]. The combination of mechanical (Al_2_O_3_ sandblasting) and chemical pre-treatment (MDP-containing primer) appeared to be particularly crucial to obtain predictable bonding to zirconia ceramics, as long as composite cement was used [13,14,15].

Since all of the aforementioned studies were in vitro, one has to investigate the durability of the SARC bonding to zirconia in the oral environment. Several studies have been performed to investigate the retentive strength of SARCs through various regimens of artificial aging. However, the results have been inconclusive. De Castro et al. [16] evaluated the bond strength of different SARCs to dentin and Y-TZP ceramic blocks before and after storage in 37 °C distilled water for 24 h and after thermal cycling (5–55 °C; 10,000 cycles). They found that aging did not significantly reduce the retentive strength between Y-TZP and dentin, regardless of the cement used. Similarly, Liu et al. [17] reported that Clearfil SA Luting, a self-adhesive resin-based luting agent containing 10-methacryloxy decyl diphosphate, had good initial and durable shear bond strength (SBS) to zirconia after 24 h and 5000 thermal cycles. Qeblawi et al. [18] tested the SBS of composite resin rods cemented to zirconia plates by two different SARCs before and after aging in moist storage conditions with 10,000 thermocycles for 24 h and 30 days. They reported that the aging condition significantly affected the bond strength to zirconia, with the highest bond strength obtained in the 24 h group. Da Silva et al. [19] evaluated the retentive strength of Y-TZP ceramic plates with different surface treatments and resin cements (one adhesive cement and one SARC) after storage in distilled water at 37 °C for either 24 h or 6 months. They demonstrated that the retentive strength of the SARC cemented to Y-TZP significantly decreased after 6 months of aging in water, irrespective of the surface treatment. Several authors have pointed to the fact that long-term water storage should be used to evaluate the durability of the bond between resin cements, including SARCs, and zirconia. A minimum incubation period of six months is advocated [20,21]. Combined long-term water storage and thermocycling are well-established artificial aging methods, which are mandatory to test the bond strength durability of SARCs to zirconia [22,23].

The disadvantage of all the aforementioned studies is the failure to simulate the clinical environment since Y-TZP ceramic plates or blocks were used as samples, instead of evaluating the bond strength between the Y-TZP crown-coping and prepared natural tooth abutments. Ehlers et al. [24] used a different model that simulated oral conditions more accurately. They tested the retentive strengths of zirconia crowns cemented to extracted human molars by different types of cements, including SARCs, after thermocycling (×5000, 5 °C/55 °C) with or without one year of water storage. They reported that SARCs did have significantly higher retentive strengths than the glass ionomer and zinc phosphate cement, but the one-year water storage had no significant effect. A drawback of this study was that all the samples went through thermocycling before the incubation period. In contrast, the retention strength of Lava Y-TZP crowns after severe aging conditions (nine months water storage and 10,000 thermocycles) yielded unsatisfactory values (<1.4 MPa) in SARCs cemented to prepared human molars [6]. The disadvantage of this study was that the intaglio surfaces of the Y-TZP crowns were not sandblasted and primed.

To the best of our knowledge, studies documenting the retentive strength of Y-TZP crowns cemented by SARCs associated with mechanical conditioning and chemical promoters, immediately after cementation and after a long-term water incubation period and thermocycling, are lacking.

Therefore, the aim of the current study is to determine the retentive strength of Y-TZP crown-copings treated by mechanical conditioning and a chemical promoter and cemented by four types of SARCs to natural prepared teeth, before and after six months of aging and thermocycling. The null hypothesis tested is that aging does not influence the retentive strengths of Y-TZP crown-copings, regardless of the type of SARC used.

## 2. Experimental Section

One-hundred-twenty freshly extracted, caries-free, intact human molars were collected for the study and stored in tap water at room temperature. The study protocol was approved by the University Institutional Ethical Committee (# 14574469). The patients were informed about the study and consented to the use of their extracted teeth. For retention purposes, the roots of all teeth were notched by a diamond bur (C1, Strauss, Ra’anana, Israel) and vertically embedded by a custom-designed alignment apparatus. The teeth were suspended in the middle of a polytetrafluoroethylene (Teflon) ring and mounted in a polymethyl methacrylate resin (Quick Resin, Ivoclar Vivadent, Schaan, Liechtenstein) 2 mm apical to the cementoenamel junction (CEJ). At all times, the teeth were kept moist (owing to the storage conditions in tap water at room temperature).

All teeth were prepared according to a standardized protocol, as previously described [15,25]. The areas of the occlusal and axial surfaces of each prepared tooth were measured according to Pilo et al. [25].

**Figure 1:**
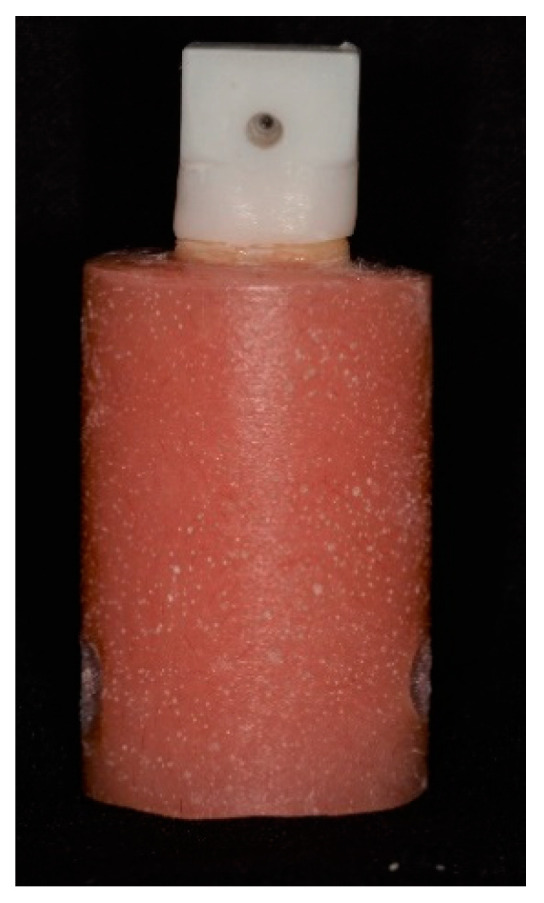
Zirconium oxide coping showing the design of the occlusal surface to enable removal post-cementation.

The prepared teeth were digitally scanned by a laboratory scanner (3Shape E1 Scanner, 3Shape A/S, Copenhagen, Denmark). The CAD-CAM zirconia copings were designed with a 0.5-mm axial and 1.0 mm occlusal thickness, a virtual spacer layer of 50-µm thickness 0.5 mm short of the margins and a loop (5 mm outer diameter and 2 mm inner diameter) extending coronally from the occlusal surface to facilitate tensile loading (Figure 1).

Following the design, 120 crown-copings of Lava^TM^ zirconia blocks (3M ESPE, Seefeld, Germany) were milled at a commercial dental laboratory (N.D.M Dental Solution, Bnei-Brak, Israel) using CAD-CAM technology (Yenadent DC40, Yenadent Ltd. Sti., Istanbul, Turkey).

The 120 prepared teeth were randomly assigned to four groups (4 × 30) according to the SARC type used: RelyX U-200 (RU200; 3M ESPE, Seefeld, Germany), G-Cem LinkAce Automix (GCA; GC, Alsip, IL, USA), TheraCem (Bisco Dental, Schaumburg, IL, USA) and Panavia SA (PAN; Kuraray Dental Co Ltd., Osaka, Japan). These 4 materials were chosen because they represent widely used and extensively studied SARC brands.

Prior to cementation, the intaglio surfaces of all crowns were sandblasted by alumina particles (Al_2_O_3_, 30 µm, 2.5 bar, 20 s) and then coated with a single layer of Z-Prime™ Plus (Bisco Dental, Schaumburg, IL, USA), according to manufacturers’ instructions, in order to promote adhesion. The cements were mixed according to the manufacturer’s recommendations. Each coping was cemented to a tooth in a standardized manner under a constant load of 50 N (Force gauge, FG 20, Lutron, Taiwan) for 10 min.

Post-cementation, specimens from each cement group were divided into two subgroups based on whether or not they were subjected to aging conditions (2 × 15). In the aged group, the cemented crown–tooth assemblies were stored in tap water at 37 °C for 6 months, followed by thermal cycling between water temperatures of 5 °C and 55 °C for 10,000 cycles, which is equivalent to an entire year of clinical physiological aging [26], with a 10 s dwell time (Y. Manes, TA, Israel). Thereafter, those crown–tooth assemblies were subjected to dislodgment force. In the non-aged group, which served as the control group, the crown–tooth assemblies were subjected to dislodgment force 24 h post-cementation (100% humidity).

**Figure 2:**
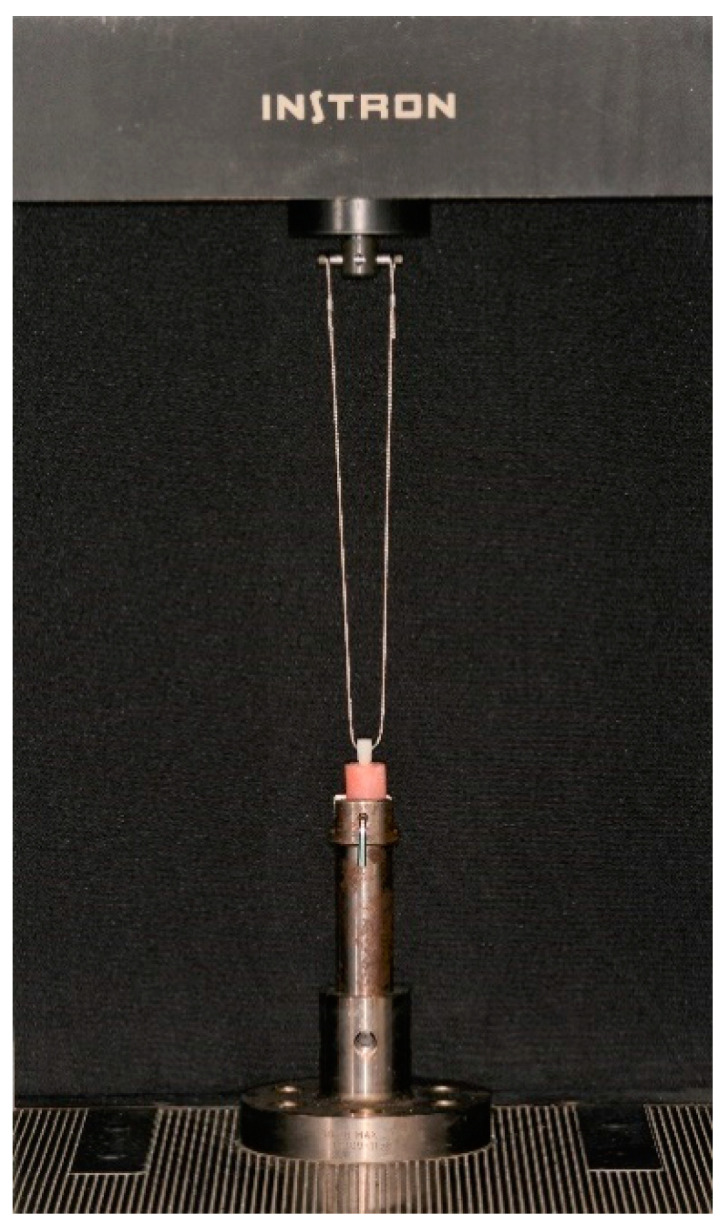
Metal cable connecting the zirconium oxide coping and the universal testing machine.

For the dislodgment force, a universal testing machine (Instron, Model 4502, Instron Corp., Buckinghamshire, UK) was used with a 1.2 mm coated diameter metal cable, which was entangled through a hole in the occlusal rectangular extension along the apico-occlusal axis, at a crosshead speed of 1 mm/min until failure (Figure 2).

The retention value (MPa) was calculated by dividing the force at dislodgment with the total surface area of each prepared sample.

The debonded surfaces of the teeth and crowns were examined with magnifying glasses at 3× magnification (Orascoptic, Middleton, WI, USA). Each matched surface of the dentin–crown-coping interface was separately analyzed (buccal, lingual, mesial, distal and occlusal). Failure was classified according to the criteria presented in Table 1.

The term “mainly” in criteria for a specific matched interface denotes that at least 75% of the interface corresponds to the description of the criterion. For each category, the number of surfaces was counted and presented as a percentage of all the surfaces analyzed for the specific cement type.

### Statistical Analysis

The data for the pre-aging and post-aging retentive strength were analyzed using a two-way analysis of variance (ANOVA); independent variables were cement type (*n* = 4) and aging process (*n* = 2). One-way ANOVA was used to analyze the difference in pre-aging and post-aging retentive strengths between the cements. The post hoc Tukey test was applied for multiple comparisons. The level of significance was set to *p* ≤ 0.05.

## 3. Results

The pre- and post-aging retentive strengths (mean ± SD) of the four types of SARCs are presented in Table 2. Two-way analysis of variance revealed that the aging treatment did not affect the retentive strength of the Y-TZP crown-copings (*p* = 0.918). Additionally, no significant differences (although borderline) were observed between the cement groups (*p* = 0.072). Nevertheless, the interaction between cement and aging treatment was statistically significant (*p* = 0.024); therefore, one-way ANOVA was used to analyze the differences between the retentive strengths of the cements in the pre- and post-aging period separately. The results showed no significant differences between the four types of SARCs in terms of the pre-aging retentive strength (*p* = 0.776) but a strong statistically significant difference for the post-aging retentive strength (*p* = 0.006).

The post hoc Tukey test for the post-aging retentive strength revealed a statistically homogeneous subset for U-200, Theracem and G-Cem as well as between Theracem, G-Cem and Panavia, implying that the only significant difference found was between the RelyX U-200 and the Panavia post-aging retentive strength (Table 3).

**Figure 3:**
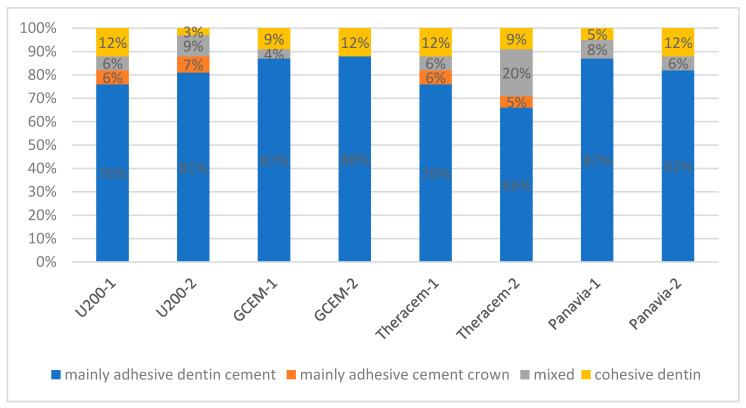
Distribution of failure modes (percentage of surfaces) for each SARC in the non-aged (1) and aged (2) groups.

Examination of the failure mode after the dislodgment of the crowns showed that in both the aged and non-aged tooth–crown failure assemblies, the dominant failure mode was mainly adhesive between the cement and dentin (range 66–87% of the surfaces), followed by mixed mode (range 4–20% of the surfaces), cohesive within the dentin (range 3–12% of the surfaces) and adhesive between the cement and crown (range 5–7% of the surfaces) (Figure 3). The only significant difference found in the retentive strength between RelyX U-200 and Panavia post-aging corresponds to more frequent cohesive dentin failures in Panavia (12%) as compared with that in RelyX U-200 (3%) and fewer adhesive cement–crown failures (0% in Panavia and 5% in RelyX U-200).

## 4. Discussion

Numerous studies have shown that improved bond strength of zirconia to composite cements can be achieved by conditioning the zirconia surface through combined mechanical and chemical pre-treatment techniques [3,5,22,27]. The main advantages of this combined technique are the chemical bonding of coupling agents with zirconia and the surface-retained particles, as well as the mechanical interlocking of the luting agents on the rough surface, following polymerization and shrinkage. This combination overcomes the deficiencies of using solely chemical bonding or grit-blasting [27]. Accordingly, the current study investigated the influence of aging on the retentive strength of Y-TZP crowns cemented by SARCs after sandblasting with alumina particles and priming with Z-Prime™ Plus. Clinical judgment should be primarily based on long-term cohort clinical studies; however, such studies are lacking, and therefore, laboratory in vitro studies are relied upon. In these circumstances, studies simulating the clinical situation and the oral conditions are preferred; however, studies that combine samples of Y-TZP-treated crowns cemented to prepared natural human teeth and not to Y-TZP ceramic plates or blocks are lacking. Testing the influence of aging by using aged cylinders of resin cements built on the ceramic plate’s surfaces and submitted to a shear bond strength test has severe limitations. The ratio between the cavosurface area exposed to the aqueous media and the total sample area is considerably higher in these experimental configurations as compared with crowns cemented to tooth dentin, thus leading to the deterioration of the bond strength, which is not necessarily observed in a clinical setup configuration. Moreover, the interface between the cement and the dentin is ignored using these methods. Such studies previously indicating deterioration of the bond strength of SARCs to Y-TZP due to aging can thus be misleading [19,28,29].

Our results demonstrate that the aging treatment did not affect the retentive strength of the Y-TZP crown-copings, nor did the type of cement. Nonetheless, the null hypothesis tested was only partially confirmed because the interaction between the type of cement and aging process was statistically significant, implying that a relationship between the retentive strength supplied by the different types of SARCs and the aging process does exist. Accordingly, a significant difference between the retentive strength of RelyX U-200 and Panavia SA post-aging was noted.

The current results, which show that the aging treatment did not affect the retentive strength of Y-TZP crown-coping regardless of the cement used, correspond well to previous reports conducted on Y-TZP blocks [16,17], as well as on zirconia crowns cemented to extracted human molars [24]. In all aforementioned studies, only mechanical treatment of the Y-TZP blocks or crowns was applied without chemical treatment, and the incubation periods were either short—24 h [17] and 60 days [16]—or long (one year) [24]. A minimum incubation period of six months for testing the bond strength durability of SARCs to zirconia is advocated, whereas shorter incubation periods might provide misleading results [20,21]. A long-term aging process (nine months water storage and 10,000 thermocycles) was also applied in our previous study conducted with an identical study design [6], but in contrast to the current study, it reported unsatisfactory retention strength values of the Lava Y-TZP crowns, which greatly differed between the SARCs. RelyX U-200 and Panavia yielded 0.49 and 0.74 MPa in the previous study, respectively [6], and 2.29 and 3.06 MPa in the current study, respectively. The difference lies in the surface treatment of the Y-TZP crowns: in the current study, we used combined mechanical (sandblasting by alumina particles) and chemical (Z-Prime™ Plus) surface treatments, while Pilo et al. [6] did not use any surface treatment. Although not all manufacturers of SARCs include recommendations for the surface treatment of Y-TZP, according to these findings and other publications [15,30], it is strongly recommended.

The main constituents of SARCs are (a) aromatic and aliphatic dimethacrylate monomers, (b) acidic methacrylate monomers to adhere to dentin and copolymerize with crosslinking monomers, (c) glass filler particles to neutralize residual acidic monomers, (d) conventional silanated filler particles to provide strength, (e) appropriate catalysts to provide the dual-cure mechanism and (f) pigments [30]. Failure analysis of the debonded specimens, both in the aged and non-aged groups, revealed that the dominant mode was mainly adhesive between the cement and dentin, implying that the bond between the cement and the zirconia was stronger. These findings correspond well to our previous study [6], in which the mode of failure between SARCs and dentin in an identical study design configuration, but without any surface treatment of the Y-TZP, was mainly adhesive between the cement and crown for the groups with the lowest retentive values (RelyX U200 and Panavia 21) and adhesive between the cement and dentin for the groups with the highest retentive values (G-Cem and Smart-Cem 2) [6]. This re-emphasizes the value of the combined mechanical and chemical treatment of Y-TZP.

Z-Prime Plus forms carboxylate and phosphate salts on sandblasted Y-TZP, thus promoting chemical adhesion [15]. Establishment of a chemical bond between Z-Prime Plus and zirconia substrates produces stable interfaces because of the higher bond energies involved compared with those in secondary bonding (H-bonding, dipole interactions, dispersion forces, etc.) [15]. In the current study, the Panavia SA post-aging retentive strength was slightly higher after six months of aging in comparison with the initial one (*p* = 0.046), while RelyX U-200 was slightly lower (*p* = 0.061), and a significant difference was found between the RelyX U-200 and the Panavia post-aging retentive strength.

While it is doubtful that these differences are of any clinical significance, they correspond to previous reports. Seto et al. [31] investigated the effects of artificial aging by thermocycling on the bond strengths to zirconia of resin cements in a setup of end-to-end bonding of cylinders of zirconia. They found that most cements had lost at least half of their initial bond strengths after 10,000 thermocycles, but Panavia F2.0 had the highest overall bond strengths among all the tested materials. Panavia SA contains MDP monomer, which creates a strong chemical bond to enamel, dentin, metal alloy and zirconia, and a unique long carbon chain silane coupling agent, LCSi monomer, which creates a strong chemical bond to all mentioned surfaces, as well as resin composites, without the need for a separate primer.

Furthermore, the slight degradation of RelyX U-200 post-aging is in agreement with a previous study concerning a similar material, RelyX Unicem, showing that the micro-tensile bond strength was reduced significantly after aging (150 d and 12,000 thermocycles) in a setup of composite resin blocks that were cemented to flat human crown–dentin surfaces [32].

The type of zirconia used in the current study belongs to the second generation, termed 3Y-TZP with reduced alumina content, primarily used for monolithic restorations [33]. It was chosen because it is the most widely used monolithic zirconia material for dental applications [34]. Newer types of ultra-translucency (5Y-TZP) and super-translucency (4Y-TZP) zirconia materials belong to the third and fourth generations respectively. Future work on aging of SARCs should be carried out on these materials, as they gain increased popularity.

Although aging did not influence the retentive strength of 3Y-TZP crown-coping cemented by SARCs in the current in vitro study, judgment on clinical behavior must be done with caution because clinical studies reporting on medium- to long-term outcomes of monolithic zirconia restorations cemented by SARCs are still lacking. Moreover, future work should include a larger number of SARCs and longer incubation periods.

## 5. Conclusions

Within the limitations of the current study, we can conclude that no significant differences in the retentive strengths of 3Y-TZP crown-copings treated by mechanical conditioning and a chemical promoter and cemented by four types of SARCs were observed after aging. The type of cement did not affect the retentive strength either; however, post-aging, Panavia SA showed significantly higher strength than RelyX U-200.

## Figures and Tables

**Table 1:** Classification of failure criteria.

Criteria	Description
Mainly * adhesive cement–dentin	Cement principally on crown surface
Mainly * adhesive cement–crown	Cement principally on dentin surface
Cohesive cement	Cement equally distributed on all tooth and crown surfaces
Mixed mode	Adhesive and cohesive cement equally
Cohesive dentin	Fracture of the dentin

* “Mainly” denotes >75%.

**Table 2:** Mean (SD) of the retentive strength (MPa) of the zirconium oxide (Y-TZP) crown-copings for the 4 types of self-adhesive resin cements (SARC) groups pre-aging and post-aging.

Cement Type	Treatment	Sample No.	Mean Retentive Value (MPa)	Standard Deviation
U-200	1	15	2.73	0.63
	2	15	2.29	0.6
	Total	30	2.51	0.65
G-Cem	1	15	2.73	0.29
	2	15	2.82	0.52
	Total	30	2.78	0.42
TheraCem	1	15	2.58	0.7
	2	15	2.51	0.49
	Total	30	2.55	0.59
Panavia	1	15	2.6	0.31
	2	15	3.06	0.8
	Total	30	2.83	0.64
Total	1	60	2.66	0.51
	2	60	2.67	0.67
	Total	120	2.67	0.59

Treatment: 1—non-aged group; 2—aged group.

**Table 3:** The post hoc Tukey test for multiple comparisons for samples that underwent 6 months of aging *.

Cement Type	N	1	2
U-200	15	2.29108	
TheraCem	15	2.51374	2.51374
G-Cem	15	2.82515	2.82515
Panavia	15		3.06259

* The materials in each subset (1 and 2) have statistically non-significant differences.
